# Low testosterone is associated with steatosis in the male population with spinal cord injury

**DOI:** 10.20945/2359-4292-2024-0047

**Published:** 2025-03-24

**Authors:** Fernanda Barros Viana Coelho, José Tadeu Stefano, Claudia Pinto Marques Souza de Oliveira

**Affiliations:** 1 Rede SARAH de Hospitais de Reabilitação, Unidade de Reabilitação de Lesão Medular, Brasília, DF, Brasil; 2 Departamento de Gastroenterologia e Nutrologia, Faculdade de Medicina da Universidade de São Paulo, São Paulo, SP, Brasil; 3 Serviço de Gastroenterologia e Hepatologia, Hospital das Clínicas (LIM 07) da Faculdade de Medicina, Universidade de São Paulo, São Paulo, SP, Brasil

**Keywords:** Spinal cord, MASLD, testosterone

## Abstract

**Objective:**

To determine the prevalence of metabolic dysfunction-associated steatotic liver disease (MASLD) in men with spinal cord injury (SCI) and to identify the independent factors associated with steatosis.

**Subjects and methods:**

This is a cross-sectional study. Inclusion criteria were males over 18 years of age with chronic traumatic SCI enrolled in a rehabilitation programme. A multivariable logistic regression model was constructed using a stepwise variable selection procedure.

**Results:**

After exclusion criteria, 160 participants were included. The prevalence of MASLD was 19%. Men with hepatic steatosis were older, had a higher prevalence of hypogonadism, obesity and diabetes mellitus. Additionally, they exhibited significantly higher levels of ALT, AST and GGT. The mean time since the SCI was 7.8 years in those with steatosis and 4.3 years in those without. Multiple logistic regression analysis revealed age (p < 0.001 OR: 1.07), time since SCI (p = 0.025, OR: 1.11) and total testosterone ≤ 300 ng/dL (p = 0.036, OR: 3.35) as significant independent variables associated with steatosis.

**Conclusion:**

Our findings suggest a prevalence of MASLD in men with SCI similar to that of the general population. Independent factors associated with steatosis were age, time since injury and total testosterone. Notably, individuals with testosterone levels ≤ 300 ng/dL were 3.35 times more likely to have steatosis compared to those with higher testosterone levels.

## INTRODUCTION

### Spinal cord injury and hepatic steatosis

Individuals with spinal cord injury (SCI) have a significantly higher 5-year incidence of cardiometabolic morbidities (56.2% *vs.* 36.4%) compared to adults without SCI (^1^). In this population, metabolic syndrome prevalence ranges from 31% to 72%, while fatty liver prevalence varies between 20 and 50% (^2,3,4^).

Spinal cord injury is believed to chronically contribute to hepatic steatosis (^3^). After SCI, there is a reduction in total lean mass by 9.5% at six months, and lean mass in the lower limbs decreases by up to 15.1% after one year (^5^). Furthermore, these patients are at the lower end of the physical activity spectrum, as determined by reduced oxygen consumption during peak physical exercise (^6^).

Anabolic hormones also appear to play a role in fatty liver disease. A study involving 55 men found that the risk of metabolic dysfunction-associated steatotic liver disease (MASLD) increased by 1% for each 1 ng/dL decrease in total testosterone and by 3% for each 1 pg/mL decrease in free testosterone, after adjusting for age, body mass index (BMI), homeostasis model assessment of insulin resistance, triglycerides (TGL), and physical activity (^4^).

Chronic inflammation’s pathogenesis may also involve changes in intestinal permeability, leading to bacterial translocation, and alterations in the pattern of intestinal microbiota. The breakdown of tight junctions in intestinal epithelial cells persists for 4 weeks after SCI in mice and for at least 8 weeks in rats (^3^). The autonomic nervous system is also implicated in the pathogenesis of hepatic steatosis in individuals with SCI.

When SCI occurs above the sixth thoracic nerve root (resulting in quadriplegia or paraplegia above T6), there is a disruption of the descending control that normally inhibits neurons of the sympathetic nervous system, potentially leading to sympathetic discharges depending on the stimulus (^3,7^). Sympathetic discharge to the liver induces glycogenolysis and gluconeogenesis, promotes a reduction in blood flow and an increase in hepatic arteriolar resistance, leading to reflex sympathetic renal activation, activation of the renin-angiotensin-aldosterone system, and consequent sodium retention.

Additionally, sympathetic stimulation of the pancreas decreases insulin production and increases glucagon production. There is also skeletal muscle vasoconstriction, associated with reduced glucose uptake, insulin resistance, and increased lipolysis. The resulting lipolysis elevates circulating free fatty acids and TGL levels, supplying more lipids to the liver and leading to increased visceral deposition (^7^).

The alteration in lean mass pattern, low level of physical activity, reduction of anabolic hormones, bacterial translocation, dysbiosis, and autonomic system dysfunction collectively contribute to an increase in adipose tissue, a reduction in lean mass, and an increase in insulin resistance (^3,6^).

### Metabolic dysfunction-associated steatotic liver disease

In 2023, a multisociety consensus redefined nonalcoholic fatty liver disease (NAFLD) terminology as metabolic dysfunction-associated steatotic liver disease (MASLD). With the name change came revisions to the disease definition.

The diagnostic criteria now include hepatic steatosis identified through imaging or biopsy, coupled with the presence of at least one of five cardiometabolic risk factors (^8^):

1. BMI ≥ 25 kg/m^2^ or waist circumference > 94/80 cm for men and women, respectively, or the equivalent adjusted for ethnicity.
2. Blood pressure ≥ 130 × 85 mmHg or specific antihypertensive drug treatment.
3. Plasma TGL ≥ 150 mg/dL or use of lipid-lowering medication.
4. Plasma high-density lipoprotein-cholesterol (HDL) ≤ 40 mg/dL for men and ≤ 50 mg/dL for women, or the use of lipid-lowering medication.
5. Fasting serum glucose ≥ 100 mg/dL or 2-hour post-load glucose levels ≥ 140 mg/dL, glycated hemoglobin (HbA1c) ≥ 5.7%, or type 2 diabetes or treatment for type 2 diabetes.

To our knowledge, no studies have investigated MASLD in individuals with SCI.

## SUBJECTS AND METHODS

This is a cross-sectional study conducted from January 2021 to January 2022. The study population comprised 191 SCI male patients admitted to the rehabilitation program at Hospital SARAH Centro, part of the SARAH Network of Rehabilitation Hospitals, in Brasília (Brazil). Ethical approval for the study was obtained from the Ethics and Research Committee of Hospital SARAH (CAAE no. 37010320.0.0000.0022).

All participants provided written informed consent before participating in the study, with assurances of voluntary participation and the option to withdraw at any time without consequence. Confidentiality regarding patient information was strictly maintained throughout the study. Inclusion criteria included male individuals aged 18 years or older with traumatic SCI lasting at least one year (classified as chronic injury).

Exclusion criteria were individuals under 18 years of age, those with less than one year of injury duration, and non-traumatic causes of SCI. The participants underwent a comprehensive assessment, including detailed medical history regarding the trauma, lifestyle habits, comorbidities, and medication use, particularly hepatotoxic drugs.

As routine in the service, participants were screened for the four primary complications associated with SCI: neurogenic bladder, neurogenic bowel, neuropathic pain, and spasticity. Assessment of neurogenic bladder involved a urodynamic study. Neurogenic bowel, neuropathic pain, and spasticity were assessed through medical history-taking and physical examination.

Measurements of weight and height were obtained using a specific method for individuals with SCI: participants were first weighed while seated in their wheelchair, followed by weighing the wheelchair alone. The patient’s weight was then calculated as the total weight minus the weight of the wheelchair. Height measurements were taken while participants were in the supine position.

Participants underwent fasting blood collection on the first day of the rehabilitation program for a range of laboratory tests, including HbA1c, HDL, TGL, low-density lipoprotein (LDL), alanine aminotransferase (ALT), aspartate aminotransferase (AST), gamma-glutamyl transferase (GGT), fasting blood glucose, and fasting total and free testosterone. Additionally, screening for diseases such as HIV, hepatitis B, and hepatitis C was conducted. Abdominal ultrasound (US) was performed by experienced radiologists using the same machine at our hospital.

The level of SCI and classification as complete (AIS A) or incomplete were defined according to the American Spinal Injury Association (ASIA) (^9^). The diagnosis of type 2 diabetes mellitus was established based on fasting blood glucose ≥ 126 mg/dL, 2-hour post-load glucose levels ≥ 200 mg/dl, or HbA1c ≥ 6.5%. Dyslipidemia was diagnosed by HDL ≤ 40 mg/dL for men and ≤ 50 mg/dL for women, serum TGL ≥ 150 mg/dL, or LDL ≥ 160 mg/dL. Systemic arterial hypertension was defined as blood pressure ≥ 140 × 90 mmHg.

In the population with SCI, BMI ≥ 22 kg/m^2^ is the most accepted cutoff for obesity, utilized both in the multivariate analysis and for diagnosing MASLD. Although no study on MASLD in the SCI population exists, we applied the MASLD criteria similarly to the general population: one cardiometabolic factor associated with an image of steatosis.

Alcohol intake was considered present if individuals reported consuming at least 210 g of alcohol per week. Those who had abstained from alcohol for more than a year were classified as abstainers. Smoking was quantified in packs/year. Individuals who had ceased smoking more than 10 years prior were considered non-smokers. The practice of physical activity was assessed using the physical activity scale for individuals with physical disabilities score (^10^). Low total testosterone was defined as ≤ 300 ng/dL (^11^). The diagnostic criteria outlined by the international consensus for MASLD were applied (^8^).

### Statistical analysis

Initially, all variables underwent descriptive analysis. For quantitative variables, this analysis involved observing minimum and maximum values and calculating means, standard deviations, and medians. The absolute and relative frequencies were computed for the qualitative variables, and the means between two groups were compared using the Student t-test.

When the assumption of data normality was not met, the non-parametric Mann-Whitney test was applied. To assess the homogeneity between proportions, we employed either the chi-square test or Fisher’s exact test, depending on the context. Predictors of steatosis were identified by selecting variables with p < 0.10 in the univariate analysis, which were subsequently included in a multivariable logistic regression model. This model was adjusted using a “stepwise” variable selection process. The adequacy of the model was demonstrated using the Hosmer-Lemeshow test. All calculations were performed using R (v. 4.3), with a significance level set at 5%.

## RESULTS

Initially, 191 individuals were assessed. However, 31 were excluded due to non-traumatic causes of SCI, age younger than 18 years old, or less than one year since SCI diagnosis. As a result, 160 patients were included in the study. Participants’ ages ranged from 20 to 78 years of age (mean 38 ± 14). Among them, 33 individuals (20.6%) exhibited steatosis on abdominal ultrasound and the prevalence of MASLD was 19.4%.

Twelve participants (0.08%) tested positive for anti-HBc but none tested positive for HBsAg. No participants had positive serology for hepatitis C. One patient was HIV positive and undergoing treatment but did not exhibit fatty liver. In the group with steatosis, no participants reported alcohol intake exceeding 210 g per week.

The prevalence of diabetes mellitus was 30% in the group with steatosis and 5% in the group without steatosis (p = 0.005). The prevalence of dyslipidemia was 76% and 56% (p = 0.061) and systemic arterial hypertension was 49% and 21% (p = 0.004) in the groups with and without steatosis, respectively. Two patients reported a previous diagnosis of sleep apnea/ hypopnea syndrome, one of which was diagnosed with fatty liver on abdominal US.

The prevalence of neurogenic bladder or neurogenic bowel was 100%. For the treatment of neurogenic bladder, 94% used oxybutynin or solifenacin, while 11% used doxazosin or tamsulosin. Regarding spasticity management, 37% utilized baclofen or tizanidine. For neuropathic pain, 33% used either pregabalin or gabapentin. No patient was excluded from the study due to the use of hepatotoxic drugs.

In the univariate analysis, statistically significant differences were observed in age, ALT, AST, GGT, total testosterone ≤ 300 ng/dL, diabetes mellitus, hypertension, BMI ≥ 22 kg/m^2^, and time since trauma. However, there were no differences in physical activity, smoking, alcohol intake, dyslipidemia, level, and classification of SCI (Table 1).

**Table 1:** The characteristics of the study participants

	Liver steatosis	No liver steatosis	P
No. of participants, n	33	127	
Age, mean (SD)	48.8 (13.7)	35.8 (13)	<0.001(^1^)
Diabetes mellitus, n (%)	10 (30.3)	6 (4.7)	0.005(^3^)
Dyslipidemia, n (%)	25 (75.8)	86 (55.9)	0.061(^2^)
BMI ≥ 22 kg/m^2^, n (%)	30 (90.9)	76 (60.8)	0.002(^2^)
Hypertension, n (%)	16 (48.5)	27(21.3)	0.004(^2^)
ALT (UI/L), mean (SD)	44.1 (24.6)	32.6 (17.6)	0.004(^1^)
AST (UI/L), mean (SD)	23.4 (9.2)	20.1 (7.4)	0.04(^1^)
Total testosterone level ≤ 300 (ng/dL), n (%)	17 (51.5)	23 (18.4)	<0.001(^1^)
Free testosterone (pg/mL), mean (SD)	7.1 (3.6)	10.8 (6)	<0.001(^1^)
GGT* (U/L), mean (SD)	61.6 (45.1)	51.5 (46.1)	0.025(^1^)
Smoking, n (%)	3 (9.4)	26 (21.5)	<0.001(^1^)
Alcohol intake (>210 g/week), n (%)	0	18 (14.2)	0.053K^1^)
Physical activity (PASIPD), mean (SD)	1.5 (2)	2.9 (4.1)	0.107(^4^)
AIS A, n (%)	21 (63.6)	79 (62.7)	1(^2^)
Quadriplegia or paraplegia ≥ T6, n (%)	8 (24.2)	28 (22)	0.718(^2^)
Time since SCI, mean (SD)	7.8 (8.2)	4.3 (5)	0.023(^4^)

BMI: body mass index; ALT: alanine aminotransferase; AST: aspartate aminotransferase; GGT: gamma-glutamyl transferase; PASIPD: physical activity scale for individuals with physical disabilities; American Spinal Injury Association impairment scale; SCI: spinal cord injury.

* Variable evaluated with logarithmic transformation; (^1^) Student t-test descriptive level of probability; (^2^) Chi-square test descriptive level of probability; (^3^) Fisher’s exact test descriptive probability level; (^4^) non-parametric Mann-Whitney test descriptive level of probability.

Free testosterone was analyzed as a continuous variable. There was a significant difference in the univariate analysis (p < 0.001), with lower free testosterone levels in the group with steatosis. Nevertheless, this difference did not persist in the multivariate analysis.

The level of physical activity was assessed using the physical activity scale for individuals with physical disabilities score, which considers recreational activities, domestic activities and work-related activities (^10^). Although the group with steatosis had higher scores, the difference between the groups was not statistically significant (p = 0.107).

For the multivariate study, variables with p < 0.10 in the univariate analysis were selected. Through the “stepwise” selection process, age (p = 0.003, OR: 1.07), time since SCI (p = 0.025, OR: 1.11) and total testosterone ≤ 300 ng/dL (p = 0.036, OR:3.35) were associated with steatosis (Table 2). The Hosmer Lemeshow test showed model adequacy (p = 0.703, not significant).

**Table 2:** Parameter values estimated by the multivariate logistic regression model

Variable	Estimated parameter	p	Odds ratio	95% CI	
				Lower limit	Upper limit
Constant	-7.708	<0.001			
Age	0.068	0.003	1.070	1.025	1.123
Time since SCI (mo)	0.108	0.025	1.114	1.018	1.235
Testosterone level ≤ 300 ng/dL	1.210	0.036	3.355	1.087	10.775

SCI: Spinal cord injury; CI: confidence interval; mo: months

## DISCUSSION

This is the first study to investigate MASLD within the SCI population. The prevalence of MASLD was 19%, and the prevalence of steatosis was 21%. The prevalence of liver steatosis in the general population varies by region, affecting approximately 25% of the global population (^12^). In Brazil, a study using US as a diagnostic method found a prevalence of around 20% for hepatic steatosis in the general population (^13^).

We anticipated a higher prevalence of fatty liver in the population with SCI, given their higher prevalence of metabolic syndrome (^14^). However, our findings suggest a prevalence similar to that of the general population. This unexpected result could be attributed to several factors. First, our study participants were relatively young, with a mean age of 38 years, which could result in a lower prevalence of comorbidities. Additionally, there may have been a selection bias, as only patients admitted to the rehabilitation program were included. It is worth noting that some individuals were undergoing their second or third rehabilitation program, indicating they may have been more aware of the importance of physical activity and a healthy diet. These factors may have influenced our observed prevalence rates.

In the general population, obesity is diagnosed when the BMI ≥ 30 kg/m^2^, and overweight when BMI ≥ 25 kg/m^2^. However, for the population with SCI, a BMI ≥ 22 kg/m^2^ is the most accepted cutoff for obesity due to changes in body composition after the trauma (^2^). Overweight is one of the cardiometabolic criteria used to diagnose MASLD. Interestingly, in our sample, the prevalence of MASLD was the same whether using a BMI cutoff of ≥ 25 kg/m^2^ or ≥ 22 kg/m^2^, because there were no individuals with a BMI between 22 kg/m^2^ and 25 kg/m^2^ as the sole cardiometabolic factor. Waist circumference was not considered in our study, as it could indicate increased levels due to abdominal muscle weakness observed in SCI patients and, consequently, produce misleading results (^14^).

Recent research, including a study published in 2021, has highlighted the role of sympathetic activation in the pathogenesis of liver steatosis (^7^). We speculated that individuals with cervical lesions (quadriplegics) and paraplegics with lesions above T6 would exhibit a higher prevalence of fatty liver due to increased sympathetic discharges, resulting in greater hepatic fat deposition.

Nevertheless, our study did not find a significant difference in the prevalence of fatty liver between groups with differing levels of SCI. This finding is consistent with previous research indicating that the level of injury is not a risk factor for cardiovascular disease in individuals with traumatic paraplegia (^15^).

### Independent factors associated with steatosis

The multivariate analysis revealed a statistically significant difference for age, time since SCI, and total testosterone levels ≤ 300 ng/dL. The most important independent factor associated with steatosis was laboratory-defined hypogonadism, defined as total testosterone levels ≤ 300 ng/dL (^11^). Individuals with testosterone levels ≤ 300 ng/dL were 3.35 times more likely to have steatosis compared to those with higher testosterone levels. This finding aligns with previous studies that have demonstrated a link between low total and free testosterone levels and NAFLD in SCI patients. Androgen deficiency has been associated with increased visceral obesity. This phenomenon is attributed to the inhibition of adipogenic differentiation and the reduction of lipoprotein lipase activity in adipose tissue, both mediated by testosterone (^4^).

SCI increases the risk of developing testosterone deficiency. A cross-sectional study compared testosterone levels in young men (age < 46 years). with chronic SCI without comorbidities to age-matched men without SCI. Individuals with SCI had a 3.7-fold higher prevalence of low testosterone (^16^). Conditions associated with hypoandrogenism include incomplete sexual development, small testes, loss of body hair, decreased libido, erectile dysfunction, gynecomastia, and hot flashes (^17^). In individuals with SCI, some symptoms, particularly those related to sexual performance, are difficult to determine.

Abilmona and cols. (^18^) found that in men with SCI, low testosterone was associated with increased fat mass, elevated visceral adipose tissue, higher fasting serum insulin and glucose levels, and reduced insulin sensitivity. Their study also revealed that men with serum testosterone levels within the normal range had a 41% lower serum TGL level compared to men with low-range serum testosterone levels (p = 0.03). Similarly, our study did not identify diabetes mellitus and dyslipidemia as significant independent variables for steatosis, aligning with the results reported by Barbonetti and cols. (^4^), who found only testosterone to be a significant factor associated with steatosis in the SCI population.

In the general population, testosterone therapy has shown efficacy in enhancing erectile function, addressing low sex drive, treating anemia, improving bone mineral density, increasing lean body mass, and alleviating depressive symptoms in men diagnosed with testosterone deficiency (^19^). A clinical study involving 22 patients with SCI compared testosterone replacement over one year between 11 individuals with hypogonadism and 11 without. The result was an increase in basal metabolic rate and lean mass in the hypogonadal group by reestablishing physiological serum testosterone levels, with minimal side effects (^15^).

McLoughlin and cols. (^17^) demonstrated that the metabolic response to testosterone replacement varies depending on its serum level. The combination of testosterone patches and resistance training with functional electrical stimulation for 16 weeks in men with SCI and an average baseline testosterone level above the cutoff for testosterone deficiency increased muscle mass, strength, bone quality, and basal metabolic rate. In contrast, testosterone patches without exercise for 16 weeks produced no significant changes in these parameters. Administering testosterone patches for 12 months in men with SCI and testosterone deficiency also increased lean tissue mass and resting energy expenditure (^17^).

Although free testosterone was lower in the group with steatosis, it was not a significant independent factor following multivariate analysis. We hypothesize that there might be a decrease in sex hormone-binding globulin concentration, caused by insulin resistance and obesity-related hyperestrogenism. Further studies are necessary for a more comprehensive understanding of androgenic hormone dynamics in the SCI population.

The other independent variables were age and time since injury. The odds of developing steatosis increased by 1.07 times for each year of age and by 1.11 times for each year since the occurrence of SCI. These findings are consistent with the literature, which indicates that advanced age is associated with increased cardiovascular risk in individuals with traumatic paraplegia (^20^).

Despite our promising findings, this study had limitations, including a selection bias, as participants were drawn from a convenience sample. Since there is no well-defined waist circumference cutoff for the SCI population, it was not possible to use it as a cardiometabolic factor for diagnosing MASLD. Another limitation is that we had only one measurement of fasting total and free testosterone.

Lastly, BMI could have posed a limitation by potentially underestimating MASLD diagnosis. However, among patients that had BMI as the unique cardiometabolic factor for MASLD diagnosis, none had a BMI between 22 kg/m^2^ and 25 kg/m^2^, so it did not impact the overall prevalence of MASLD. The hypertension diagnosis cutoff for the SCI population is the same as for the general population, but it might not be appropriate due to their typically lower blood pressure.

In conclusion, this is the first study to explore MASLD in individuals with SCI. Cardiovascular diseases are the leading cause of death in this group, highlighting the importance of understanding metabolic dysfunctions to improve quality of life and long-term outcomes. Due to the unique characteristics of individuals with SCI, it is essential to develop SCI-specific diagnostic criteria for MASLD.

The factors independently associated with steatosis in males with traumatic SCI include age, time since injury, and total testosterone levels above 300 ng/dL. The effectiveness of testosterone replacement therapy remains unclear, and further research is necessary to elucidate the metabolic alterations in this population and to investigate the relationship between testosterone levels and steatosis following SCI.
